# A Mitochondrial Transcription Termination Factor, *ZmSmk3*, Is Required for *nad1* Intron4 and *nad4* Intron1 Splicing and Kernel Development in Maize

**DOI:** 10.1534/g3.119.400265

**Published:** 2019-06-13

**Authors:** Zhenyuan Pan, Xuemei Ren, Hailiang Zhao, Lei Liu, Zengdong Tan, Fazhan Qiu

**Affiliations:** National Key Laboratory of Crop Genetic Improvement, Huazhong Agricultural University, Wuhan 430070, P.R. China

**Keywords:** mitochondrial, embryo, endosperm, intron splicing, *Zea mays*

## Abstract

The expression systems of the mitochondrial genes are derived from their bacterial ancestors, but have evolved many new features in their eukaryotic hosts. Mitochondrial RNA splicing is a complex process regulated by families of nucleus-encoded RNA-binding proteins, few of which have been characterized in maize (*Zea mays* L.). Here, we identified the *Zea mays small kernel 3* (*Zmsmk3*) candidate gene, which encodes a mitochondrial transcription termination factor (mTERF) containing two mTERF motifs, which is conserved in monocotyledon; and the target introns were also quite conserved during evolution between monocotyledons and dicotyledons. The mutations of *Zmsmk3* led to arrested embryo and endosperm development, resulting in small kernels. A transcriptome of 12 days after pollination endosperm analysis revealed that the starch biosynthetic pathway and the zein gene family were down-regulated in the *Zmsmk3* mutant kernels. ZmSMK3 is localized in mitochondria. The reduced expression of *ZmSmk3* in the mutant resulted in the splicing deficiency of mitochondrial *nad4* intron1 and *nad1* intron4, causing a reduction in complex I assembly and activity, impairing mitochondria structure and activating the alternative respiratory pathway. So, the results suggest that ZmSMK3 is required for the splicing of *nad4* intron 1 and *nad1* intron 4, complex I assembly and kernel development in maize.

The expression systems of mitochondrial genes are derived from their prokaryotic ancestors, but they have evolved many new features in their eukaryotic hosts (Barkan 2011; Hammani and Barkan 2014). These new features are involved in RNA transcription, RNA editing, intron splicing, RNA maturation and RNA degradation, which are far more complex than those of their prokaryotic progenitors and regulated by a plethora of nucleus-encoded proteins. Although these families of nucleus-encoded proteins evolved independently, they seem to share a common structural organization, which is consisted of similar repeated helical motifs and can form the solenoid structures of chromatin (Hammani and Barkan 2014). These families include the pentatricopeptide repeat (PPR) proteins, the mitochondrial transcription termination factors (mTERF), the half-a-tetratricopeptide (HAT) proteins and the octotricopeptide repeat (OPR) proteins.

The mTERF proteins are widely distributed in metazoans and plants, and green alga, but apparently absent in fungi and prokaryotes (Roberti *et al.* 2009). The mTERF proteins were originally identified and characterized in the metazoans. In metazoans, There are four members of mTERF proteins (mTERF1- mTERF4), which are encoded by nuclear genes and consist of multiple mTERF motifs, each of which contains about 32 amino acids that form three X_3_LX_3_ leucine zipper-like elements (Zhao *et al.* 2014). All of mTERFs are targeted in mitochondria, and they could regulate transcription, translation and DNA replication of mitochondria genes in metazoans.(Chen *et al.* 2005; Roberti *et al.* 2009; Cámara *et al.* 2011). MOC1 (mterf-like gene of *Chlamydomonas*1) is the first mTERF characterized in a photosynthetic organism, green alga *Chlamydomonas reinhardtii* (Schönfeld *et al.* 2004). MOC1 is a mitochondrial protein, which binds specifically to a sequence within the mitochondrial ribosomal RNA (rRNA)-coding module S3 (Wobbe and Nixon 2013). The loss of MOC1 causes perturbed mitochondrial DNA (mtDNA) expression and sensitivity to high light.

In plants, there are more members of mTERFs, however, only a few mTERFs have been well functionally characterized, all of which encode chloroplast or mitochondrial proteins (Quesada 2016). BELAYA SMERT/RUGOSA2 (BSM/RUG2) is a dual-targeted mTERF protein that affects the expression levels in both the mitochondria and the chloroplasts. The *bsm* mutation causes arrested embryo development and a significant retardation in plant growth and development (Babiychuk *et al.* 2011). MTERF DEFECTIVE IN Arabidopsis1 (MDA1) functions in chloroplast development and abiotic stress responses; *mda1* mutants display pale-green pigmentation and growth retardation, enhanced salt, osmotic stress tolerance, altered sugar responses, and reduced abscisic acid (ABA) sensitivity (Robles *et al.* 2012a; Robles *et al.* 2012b). *Suppressor of hot1-4 1* (*SHOT1*) encodes a mitochondrial mTERF protein that suppresses the phenotype of the *hot1-4* mutation in *Arabidopsis thaliana*; the loss of SHOT1 function reduces plant growth and enhances thermos tolerance in the absence of HSP101 (Kim *et al.* 2012). *Singlet oxygen-linked death activator 10* (*SOLDAT10*) encodes a mitochondrial mTERF protein that suppresses the *flu* mutation; the *soldat10* mutants decrease levels of plastid-specific rRNA and attenuate protein synthesis in plastids (Meskauskiene *et al.* 2009). TWR-1/MTERF9 is a chloroplast protein, and the loss of the TWR-1/MTERF9 results in defective chloroplast development, reduced mesophyll cell numbers, paleness, and stunted growth (Robles *et al.* 2015). Arabidopsis mTERF6 is localized in both chloroplasts and mitochondria, which is required for maturation of the chloroplast transfer RNA^Ile^(GAU); the loss of *mTERF6* perturbs plastid development and results in seedling lethality (Romani and Manavski 2015). Arabidopsis mTERF15 is a mitochondrial protein required for mitochondrial *nad2* intron 3 splicing and complex I activity (Hsu *et al.* 2014). The null homozygous T-DNA *mterf15* mutants display substantial retardation of both vegetative and reproductive development (Hsu *et al.* 2014). Zm-mTERF4 is the ortholog of the Arabidopsis protein BSM/RUG2. Zm-mTERF4 localizes to the chloroplast stroma and is required for group II intron splicing and the accumulation of plastid ribosomes in maize *(Zea mays)* chloroplasts (Hammani and Barkan 2014). The *Zm-mterf4-1*, the null mutant, displays an ivory leaf phenotype, while *Zm-mterf4-2* mutant displays a pale yellow-green phenotype. These findings reveal that the plant *mTERF* genes are required for the regulation of gene expression in chloroplasts and mitochondria.

Here, we identified and characterized a candidate gene *ZmSmk3*, which is responsible for kernel development in maize. *ZmSmk3* encodes an mTERF protein targeted to mitochondria, which contains two mTERF motifs conserved in monocotyledon. The *Zmsmk3* mutation arrested the splicing of mitochondrial *nad4* intron 1 and *nad1* intron 4, reduced the assembly and activity of complex I, impaired mitochondrial structure, and increased the expression levels of the alternative oxidases AOX2 and AOX3, which resulted in small kernels. In summary, we present a new mitochondrial mTERF protein required for mitochondrial intron splicing, complex I assembly and kernel development in maize.

## Materials and methods

### Plant materials

The *Zmsmk3* mutant was isolated from the UniformMu collection stocks, No. UFMu-06341 (McCarty *et al.* 2005). WT kernels in a segregating ear were used for the evaluation of the kernel phenotypes. For the genotype analysis and the co-segregation analysis, total DNA was extracted from the leaves of each individual using the modified CTAB method.

### Cytological observation

Kernels at 6, 12, and 21 DAP were harvested from the selfed +/*Zmsmk3* heterozygotes and fixed overnight in 4% paraformaldehyde (Sigma-Aldrich, St. Louis, MO, USA), dehydrated in an ethanol gradient series (30%, 50%, 70%, 85%, 95%, and 100% ethanol), and embedded in Paraplast Plus (Sigma-Aldrich). The samples were sectioned into 8–12 µm slices using a Leica RM2265 microtome (Leica Microsystems, Wetzlar, Germany) and stained with 0.5% toluidine blue O. Images were captured using a Leica MZFLIII microscope (Leica Microsystems).

For the TEM analysis, the 10-DAP endosperms of the WT and *Zmsmk3* kernels were fixed, washed, dehydrated, embedded, and cut into ultrathin sections, as previously described (Ren *et al.* 2017). Ultra-thin sections were obtained using a Leica EM UC7 ultra microtome (Leica Microsystems). The sections were stained with uranyl acetate and subsequently with lead citrate, then imaged using a Tecnai G^2^ 20 TWIN transmission electron microscope. These procedures were performed by Pei Zhang (Core Facility and Technical Support, Wuhan Institute of Virology, China).

### RNA extraction and gene expression analysis

The developing tissues, including the root, stem, leaf, tassel, ear, silk, ovary, endosperm, and embryo, were collected to analyze the expression pattern of *ZmSmk3*. The total RNA of these tissues was extracted using the Ambion Pure Link Plant RNA Reagent (Thermo Fisher Scientific, Waltham, MA, USA), then reverse transcribed using M-MLV reverse transcriptase (Thermo Fisher Scientific) according to the manufacturer’s instructions. RT-qPCR was performed for the expression pattern analysis of *ZmSmk3* using the primer pairs ZmSmk3-qPCR-F and ZmSmk3-qPCR-R (primer sequences listed in Supplemental Table 2). The RT-qPCR was performed using the SYBR Select Master kit (Thermo Fisher Scientific), following the manufacturer’s instructions, with three biological replicates. The maize actin gene (*ZmActin*; *GRMZM2G126010*) was used as the internal control, and the relative expression levels were calculated using the comparative Ct method.

For the transcriptional analysis of the mitochondrial and *AOX* genes, the total RNAs were extracted from 12-DAP kernels of the WT and the *Zmsmk3* mutant after the pericarp was removed. The RNAs were treated with RNase-free DNase I, then their abundances were normalized to both the total RNA level and the abundance of the *ZmActin* transcripts.

The primers used for the amplification of the mitochondrial genes, RT-qPCRs, and RT-PCRs in Figure 6 and Figure S4 were previously published by Xiu *et al.* (2016).

### RNA in situ hybridization

WT kernels at 12 DAP were used for the mRNA *in situ* hybridization assay. The kernels were fixed in 4% paraformaldehyde, following the procedures for cytological observation outlined above. The probes used to detect the *ZmSmk3* transcripts corresponded to the full length of *ZmSmk3* cDNA, and were constructed using the ZmSmk3-CDS-F/R primers. The T7 and Sp6 polymerases were used to produce sense and antisense RNA probes for *in vitro* transcription, which were then labeled with digoxigenin-UTP (Roche, Basel, Switzerland). Nitroblue tetrazolium and 5-bromo-4-chloro-3-indolyl phosphate (Roche) were used to detect the digoxigenin signal, following the manufacturer’s instructions. A Nikon Eclipse 80i differential interference contrast microscope (Nikon Instruments, Tokyo, Japan) was used to the capture images, following the methods described by Ren *et al.* (2017).

### Subcellular localization of ZmSMK3

The full-length coding sequence of *ZmSmk3* without the termination codon was amplified using the ZmSmk3-Kpn1-F and ZmSmk3-Xba1-R primers, which are listed in Supplemental Table 2. The PCR product was purified, verified by sequencing, then inserted into the pM999-GFP vector to generate a fusion construct. The construct was introduced into maize protoplasts derived from seedling leaves using a polyethylene glycol (PEG)/calcium-mediated transformation (Yoo *et al.* 2007). MitoTracker Red (Thermo Fisher Scientific) was used as the mitochondrion marker.

### Mitochondrial complex activity assay and western blotting assay

Crude and intact mitochondria were isolated from maize embryos and endosperms at 15-DAP, as described by Ren *et al.* (2017). The mitochondrial suspension was used for a BN-PAGE using a Native PAGE sample prep kit (Thermo Fisher Scientific). The in-gel complex I activity assay was performed as described previously (Meyer *et al.* 2009). Gel strips were loaded with extracts from 150 µg maize mitochondria.

Crude mitochondrial extracts from 15-DAP kernels with the pericarp removed were used for the western blot analyses using antibodies (from Agrisera, Vännäs, Sweden) against Nad6 (subunit of complex I), Cytc (subunit of complex III), COX2 (subunit of complex IV), AtpB (subunit of complex V), AOX (alternative oxidase), and α-tubulin as a loading control.

### RNA-Seq analysis

Total RNA was extracted from three biological samples of 15-DAP WT and *Zmsmk3* seeds with the pericarp removed. Library construction was performed according to the standard instructions provided by Illumina (San Diego, CA, USA). FastQC (http://www.bioinformatics.babraham.ac.uk/projects/fastqc/) and Trimmomatic (Bolger *et al.* 2014) were used to obtain clean reads. The RNA-Seq data were analyzed using TopHat and Cufflinks (Ghosh and Chan 2016) and normalized as fragments per kilobase of transcript per million mapped reads (FPKM). A hypergeometric distribution was used for the statistical analysis.

### Data availability

There are two files (one excel file and one word file) in the Supplemental Material, File S1 and File S2. File S1 contains two supplementary tables. Table S1 includes Gene ontology classifications of DEGs with functional annotation. Table S2 shows Primers used in this study. File S2 contains four supplementary figures. Figure S1 showed aborted development in *Zmsmk3* mutants. Figure S2 showed identification of *ZmSmk3* by TAIL-PCR. Figure S3 showed phylogenetic analysis of ZmSMK3. Figure S4 showed RT-PCR analysis of mitochondrial tRNA and rRNA encoded genes transcriptional levels in the wild type and the *Zmsmk3* mutant. Supplemental material available at FigShare: https://doi.org/10.6084/m9.figshare.8217473.

## Results

### Embryo and endosperm development are arrested in Zmsmk3

**Figure 1:**
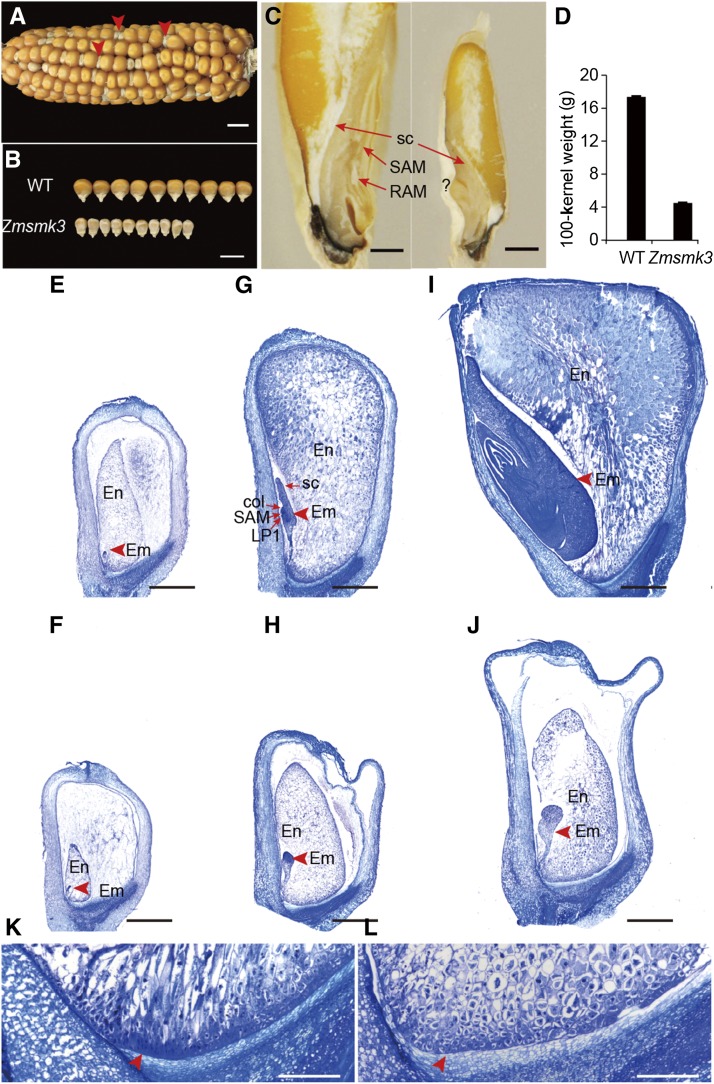
The *Zmsmk3* mutants are arrested in embryogenesis and endosperm development. (A) A mature segregating ear of maize. Arrows indicate the *Zmsmk3* mutant kernels. (B) A size comparison between the wild-type (WT) and *Zmsmk3* mutant kernels. (C) Dissection of mature WT (left) and *Zmsmk3* (right) kernels. RAM, root apical meristem; SAM, shoot apical meristem; sc, scutellum, (D) Comparison of WT and *Zmsmk3* kernel weights. Sections of developing kernels at 6 days after pollination (DAP; E-F), 12 DAP (G-H), and 21 DAP (I-J), and the basal endosperm transfer layer (BETL) at 21 DAP (K-L). (E, G, I, K) Wild-type (WT) kernels, (F, H, J, L) *Zmsmk3* kernels. col, coleoptile; em, embryo; en, endosperm; LP1, first leaf primordium; SAM, shoot apical meristem; sc, scutellum. Arrows in K and L indicate the BETL. Values are the means with SE; *n* = 3 individuals. Scale bar = 1 cm in (A, B), 1 mm in (C, E, F, G, H, I, J) and 0.2 mm in (K–L).

The *Zmsmk3* is a small kernel (*smk*) mutant identified from the Uniform Mu population of W22 plants mutated with *Mutator* transposons, stock No. UFMu-06341 (McCarty *et al.* 2005). The self-pollinated *ZmSmk3* heterozygotes segregated into normal and small kernels at a ratio of approximately 3:1 (1651:526, χ^2^-test, *P* > 0.05; Figure 1A), indicating that *Zmsmk3* is a monogenic recessive nuclear mutant allele. Compared with the kernels of wild type (WT), the tops of the mutant kernels were shrunken and the starchy endosperm did not fill the pericarp (Figure 1B and 1C). The 100-kernel weight of the mutant was about 1/4 of that of the WT (Figure 1D). During sectioning of the mature kernels, some of the mutant embryos only had a root apical meristems (RAM), (Figure S1A and S1B), some embryos were even necrotic (Figure S1C), and only part of embryos were well-structured with smaller size. (Figure 1C). The germination rate and a seedling survival rate of *Zmsmk3* were sharply decreased. About 75% of *Zmsmk3* could germinate, however, some of which developed into abnormal seedlings (Figure S1E), resulting in only 30% well-structured seedlings (Figure S1D), which showed much slower growth than the WT (Figure S1F). These results indicated that the mutation of *ZmSmk3* affected both kernel and plant development.

To assess the impact of the *Zmsmk3* mutation on the development of the embryo and endosperm, we examined different stages after pollination of the mutant and WT kernels in the same segregating ears using paraffin sectioning and microscopy. At six days after pollination (DAP), the size of *Zmsmk3* kernels were similar to those of the WT; however, the embryos and endosperms were smaller in *Zmsmk3* kernels (Figure 1E, F). At 12 DAP, the WT embryos had progressed to the late embryogenesis stage and had differentiated the first leaf primordium, coleoptile and SAM, and the starchy endosperm had filled the pericarp (Figure 1G). However, in the 12-DAP *Zmsmk3* kernels, the embryos remained at the transition stage and there was an obvious gap between the endosperm and the pericarp, which persisted in the later stages (Figure 1H). At 21 DAP, the WT kernels had developed a mature embryo with a scutellum, four leaf primordia, and a starch-filled endosperm (Figure 1I), while the development of the *Zmsmk3* kernels remained arrested (Figure 1J). We further compared the differentiation of the basal endosperm transfer layer (BETL) in the WT and *Zmsmk3* endosperm at 21 DAP. In the WT, there were cell wall ingrowths in the BETL cell layers, which could increase the surface area of the BETL plasma membrane (Figure 1K). In *Zmsmk3*, the BETL cell structure was diminished, which might impede the nutrient transport from the maternal placenta to the developing endosperm (Figure 1L). These results indicated that both embryogenesis and endosperm development were severely arrested in *Zmsmk3* mutant.

### The deposition of storage substances during seed filling is defective in Zmsmk3

**Figure 2:**
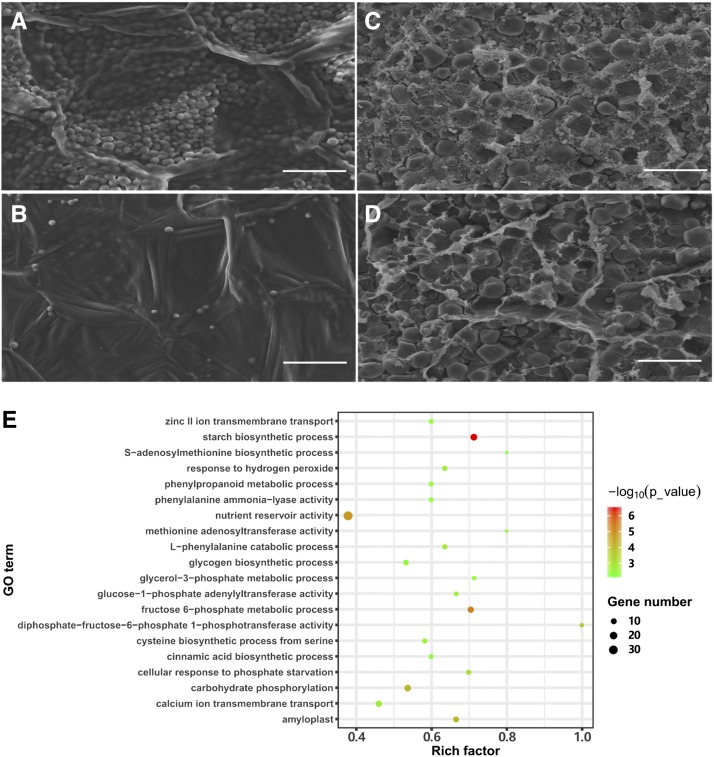
Storage substances is reduced in the *Zmsmk3* kernels. Starch accumulation in (A, B) 12 DAP kernels, (C, D) 25-DAP kernels, (A, C) wild-type kernels, and (B, D) *Zmsmk3* kernels. Scale bar = 50 µm. (E) GO analysis of differentially expressed genes (DEGs) between the wild-type and *Zmsmk3* kernels.

We analyzed the starch deposition in the endosperm of the WT and *Zmsmk3* kernels. At 12 DAP, there were many starch granules in the WT endosperm (Figure 2A) but fewer in *Zmsmk3* mutants (Figure 2B). At 25 DAP, the starch granules in the outer region of the WT endosperm were tightly embedded in a proteinaceous matrix, and endosperm cell structure had already disappeared (Figure 2C); however, in the mutant, the protein matrix was diminished and the endosperm cell structure was still retained (Figure 2D).

To better understand the impacts of *Zmsmk3*, we removed the pericarps from WT and *Zmsmk3* kernels harvested from the same segregating ear at 12-DAP and performed a transcriptome analysis using RNA-seq. Among the 44,466 genes expressed in both the WT and *Zmsmk3* kernels, we identified 5,113 significantly differentially expressed genes (DEGs) between the two genotypes using a threshold fold change > 2.0 and *P* < 0.05. Of these, 3,521 genes were upregulated and 1,592 were downregulated in *Zmsmk3*. The gene ontology (GO) classifications revealed that the DEGs were largely associated with storage filling and energy metabolism (Figure 2E). DEGs related to starch biosynthesis (GO: 00019252, *P* = 3.47E-07), nutrient reservoir activity (GO: 0045735, *P* = 2.05E-05), and amyloplast function (GO: 0009501, *P* = 8.80E-05) were extensively downregulated (Supplemental Table 1). The DEGs related to fructose 6-phosphate metabolism (GO: 0006002, *P* = 6.73E-06) and carbohydrate phosphorylation (GO: 0046835, *P* = 9.77E-05) were generally upregulated. These results indicated that the pathways of storage product deposition and energy harvesting were greatly affected in *Zmsmk3*.

### Identification of the candidate gene for Zmsmk3 by TAIL-PCR

**Figure 3:**
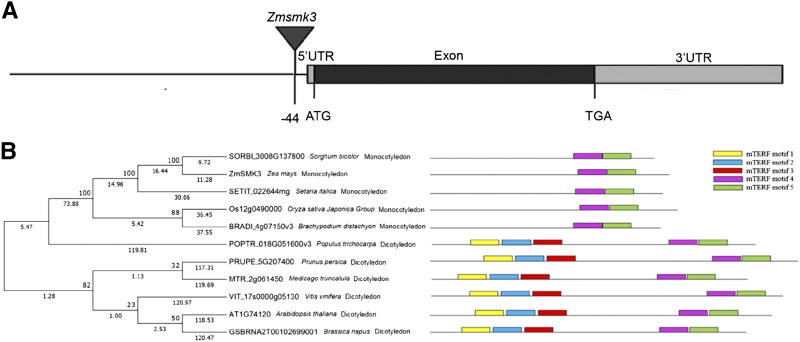
Identification of the candidate gene for *Zmsmk3* . (A) Gene structure of the candidate gene for *Zmsmk3*. The location of the insertion in the candidate gene *Zmsmk3* is indicated by the triangle. (B) Phylogenetic analysis and mTERF motifs alignment of candidate gene ZmSMK3 with its orthologs. (Left) Phylogenetic analysis of the candidate gene ZmSMK3. ZmSMK3 and identified homologous proteins in *Sorghum bicolor*, *Setaria italica*, *Oryza sativa Japonica Group*, *Brachypodium distachyon*, *Populus trichocarpa*, *Prunus persica*, *Medicago truncatula*, *Vitis vinifera*, *Arabidopsis* and *Brassica napus* were aligned by ClustalW. The phylogenetic tree was constructed using MEGA7 by Neighbor-joining method. The numbers at the nodes represent the percentage of 1000 bootstraps. The numbers at the lines represent the branch lengths. (Right) The alignment of the ZmSMK3 protein with its orthologs. mTERF motifs are indicated with different colors.

To identify the causal gene of the *Zmsmk3* mutant, Genomic DNA extracted from leaves of *Zmsmk3*/+ and +/+ individuals, whose genotype had been ascertained by selfing, was used for modified thermal asymmetric interlaced PCR (TAIL-PCR) (Settles *et al.* 2004; Zhang *et al.* 2013). For this procedure, three arbitrary primers combined with specific *Mu*-flanking primers were used to amplify larger flanking sequences from *Mu* element insertions, and the specific DNA binds (Figure S2A-C) for *Zmsmk3* were identified and sequenced. The results showed that the *Mu* element was inserted into the promoter of gene *GRMZM2G177019*, 44 bp upstream of the predicted translation start site (ATG) in *Zmsmk3* mutants. Co-segregation analysis was performed on a total of 500 F_2_ individuals from the selfing heterozygotes, and the results showed that the *Mu* element insertion was tightly linked to *Zmsmk3* without any recombination (Figure S2D). So *GRMZM2G177019* is the key candidate gene for *Zmsmk3. GRMZM2G177019* (*ZmSmk3*) is an intron-free gene encoding an mTERF protein (Figure 3A). Phylogenetic analysis was conducted with 143 homologous proteins of ZmSMK3 from six species (*Oryza sativa*, *Zea mays*, *Arabidopsis*, *Sorghum bicolor*, *Setaria italic*, and *Brachypodium distachyon*), and the results showed that ZmSMK3 was highly homologous to mTERF15 (AT1G74120) (Figure S3), which is required for the splicing of *nad2* intron 3 in *Arabidopsis thaliana* (Hsu *et al.* 2014). However, when the species for phylogenetic analysis was enlarged to eleven (Figure 3B), including both monocotyledons and dicotyledons, two branches were found in the phylogenetic tree for monocotyledons and dicotyledons respectively. Interestingly, by aligning the homologous proteins of the eleven species, we found that there were five mTERF motifs (http://smart.embl-heidelberg.de/) in the dicotyledonous species; but only two mTERF motifs are included in the monocotyledonous species (Figure 3B).

### ZmSmk3 is constitutively expressed and ZmSmk3 protein is targeted to mitochondria

**Figure 4:**
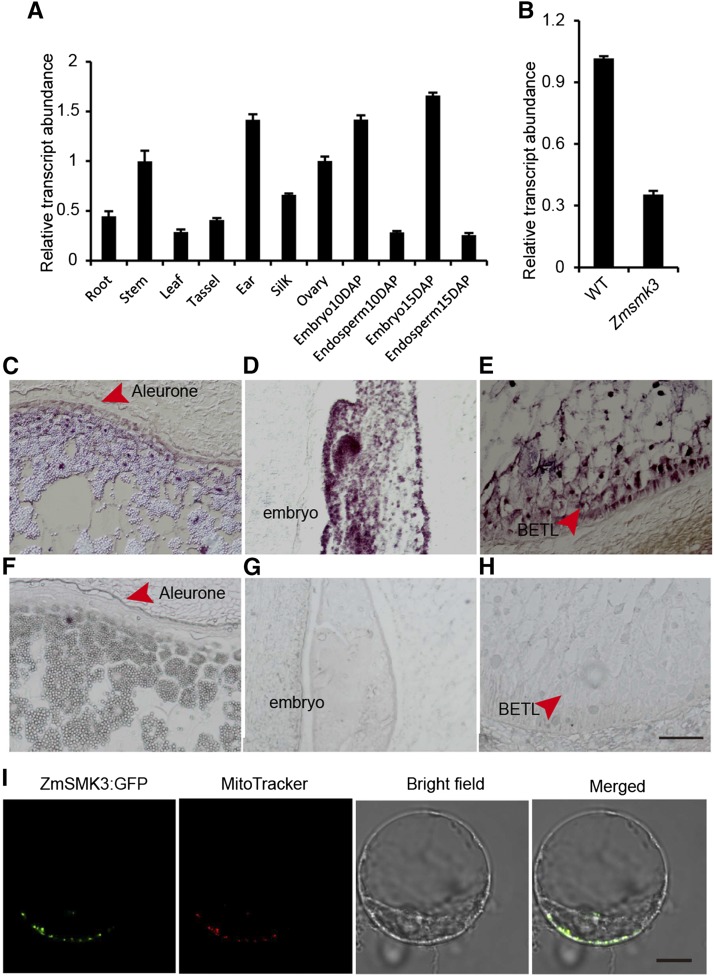
The expression of *ZmSmk3* (A) Expression profiles of *ZmSmk3* in various tissues. (B) Comparison of *ZmSmk3* expression in the wild-type (WT) and *Zmsmk3* mutant kernels. RNA was extracted from kernels at 12 days after pollination (DAP) following the removal of the pericarp. For each RNA sample in (A) and (B), three technical replicates were performed. Values are means with SE; *n* = 3 individuals. (C–H) mRNA *in situ* hybridization analysis of *ZmSmk3* in 12-DAP WT kernels using an antisense probe (C–E) and a sense probe (F–H). (I) Subcellular localization of ZmSMK3. The ZmSMK3:GFP fusion protein was transiently expressed in maize leaf protoplasts. Fluorescent signals from ZmSMK3:GFP are displayed in green and mitochondria stained with MitoTracker are red. Scale bar = 100 µm in (C–H) and 10 µm in (I).

Quantitative real-time PCR (RT-qPCR) was performed to identify the expression pattern of *ZmSmk3*. As a result, *ZmSmk3* was ubiquitously expressed in all tested tissues, with higher expression levels in the stem, ear, ovary, and embryo and lower levels in the root, leaf, tassel, silk, and endosperm (Figure 4A). We next genotyped developing seeds with the pericarp removed. Homozygous *Zmsmk3* seeds at 12-DAP with the pericarp removed were used to analyze expression of *ZmSmk3* and mitochondrial genes. Transcript levels were reduced in the *Zmsmk3* homozygous mutants (Figure 4B). To further characterize *ZmSmk3* spatial expression of the developing kernels, an mRNA *in situ* hybridization was performed in the kernels at 12 DAP. The hybridization signal was detected in the aleurone layer (Figure 4C), the BETL of the endosperm (Figure 4D), and the SAM of the embryo (Figure 4E), with particularly high expression levels in the BETL and SAM. Hybridization to the sense RNA probe did not detect any significant signals (Figure 4F-H). These results suggested that *ZmSmk3* was ubiquitously expressed in the developing kernels, but may play an important role in the BETL and SAM of the developing kernels.

To determine the localization of ZmSMK3, the full-length coding region of the gene was fused to GFP and transiently expressed in maize protoplasts. The GFP signals were detected in small dots that were identified as mitochondria by observing the red fluorescence of the MitoTracker Red dye (Figure 4I). This result indicated that ZmSMK3 was targeted to the mitochondria.

### Zmsmk3 exhibits deficiency in the splicing of mitochondrial nad4 intron 1 and nad1 intron 4

**Figure 5:**
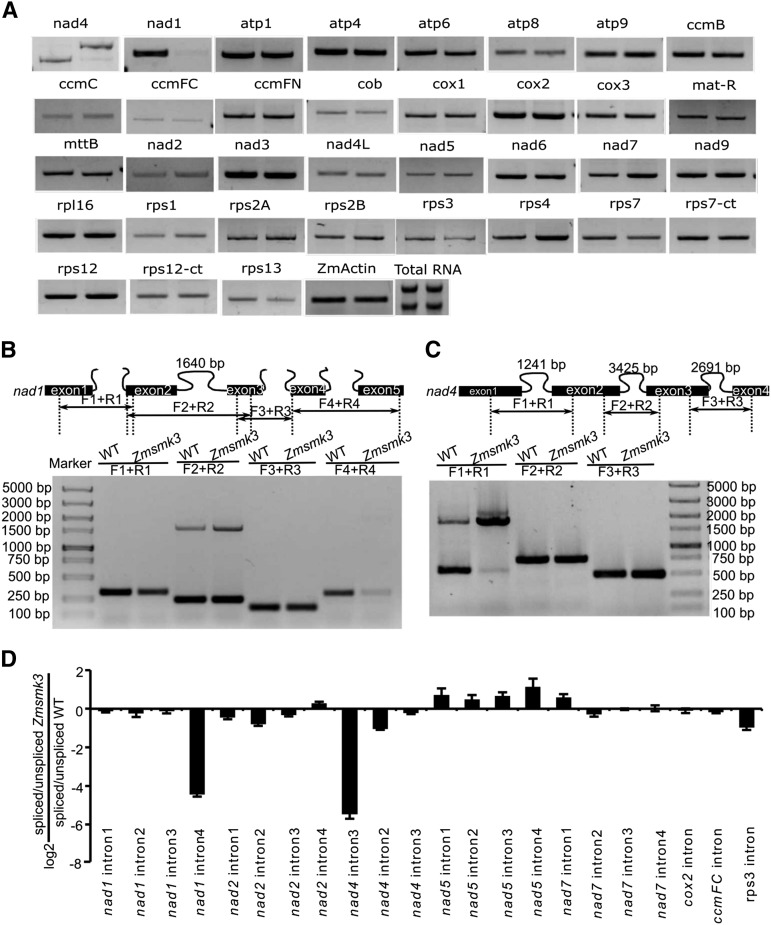
The expression of nad1 and nad4 is dramatically decreased due to the Splicing deficiency of mitochondrial nad4 intron 1 and nad1 intron 4. (A) RT-PCR analysis of 35 mitochondria-encoded transcripts in WT (left) and Zmsmk3 (right) seeds. (B) Schematic structure of the maize mitochondrial nad1 (up) and the splicing efficiency of the four introns by RT-PCR (down). (C) Schematic structure of the maize mitochondrial nad4 (up) and the splicing efficiency of the three introns by RT-PCR (down). (D) Quantitative RT-PCR analysis of 22 intron-containing mitochondrial transcripts. The RNA was isolated from the same ear segregating for WT and Zmsmk3 seeds at 12 DAP. ZmActin (GRMZM2G126010) was served as internal control.

Because ZmSMK3 appeared to target to mitochondria, we investigated whether the *Zmsmk3* mutation had any effect on gene expression in this organelle. We analyzed the transcript levels of the maize NB mitochondrial genes in wild-type and *Zmsmk3* mutants by RT-PCR. Total RNAs were extracted from 12 DAP kernels of wild-type and *Zmsmk3* from the same segregating ear with the pericarp removed. Gene-specific primers were used as Xiu described (2016) to amplify transcripts of mitochondrial genes from the cDNA template. The results showed that the expression level of most mitochondrial genes was not significantly different between wild-type and the *Zmsmk3* mutant and some tRNAs were a little higher in *Zmsmk3*, while the precursor transcript levels of *nad4* were high, and the abundance of its mature transcript was dramatically reduced, and the mature *nad1* transcripts were also dramatically reduced (Figure 5A and Supplementary Fig. S4).

Primers spanning the exons adjacent to the introns of the mature *nad1* and *nad4* transcripts were further used to examine the intron-splicing efficiency in the *Zmsmk3* and its sibling WT kernels. The results revealed that the splicing efficiency of *nad1* intron 4 and *nad4* intron 1 was decreased in the *Zmsmk3* mutant (Figure 5B-C). Furthermore, we examined the ratio of spliced to un-spliced transcripts for 22 of mitochondrial intron in *Zmsmk3* and WT using quantitative RT‐PCR (qRT‐PCR). Of the 22 introns examined, splicing efficiency of the *nad1* intron 4 and *nad4* intron 1 was dramatically decreased in *Zmsmk3* compared with that in WT (Figure 5D), but splicing efficiencies of the other introns were not significantly affected in *Zmsmk3* (Figure 5D). These results demonstrate that *ZmSmk3* is required for the splicing of *nad1* intron 4 and *nad4* intron 1.

### Zmsmk3 exhibits deficiency of mitochondrial complex I assembly and impaired mitochondrial function

**Figure 6:**
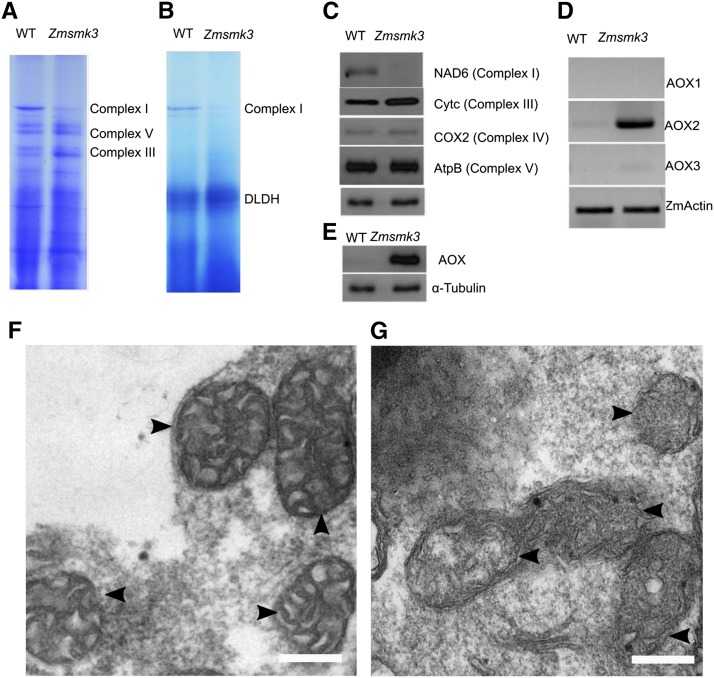
The deficiency of complex I assembly and disordered mitochondrial structure in *Zmsmk3*. (A) Comparison of the accumulation of complex I in the wild type (WT) and *Zmsmk3* mutant. Mitochondrial complexes of 12-DAP maize kernels without pericarps were separated using 3–12.5% blue native polyacrylamide gel electrophoresis (BN-PAGE). Complexes I, III, and IV are indicated. (B) Comparison of the NADH dehydrogenase activity of complex I in the WT and *Zmsmk3*. Darker staining indicates higher levels of activity. Dihydrolipoamide dehydrogenase (DLDH) was used as a loading control. (C) Comparison of the abundance of respiratory enzymes in the WT and *Zmsmk3*. Crude mitochondrial extracts from 12-DAP kernels with the pericarp removed were used for a western blot analysis using antibodies against Nad6 (subunit of complex I), Cytc (subunit of complex III), COX2 (subunit of complex IV), AtpB (subunit of complex V) and α-tubulin as a sample loading control. (D) RT-PCR analysis of *AOX1*, *AOX2*, and *AOX3* expression in the WT and *Zmsmk3* mutant. Total RNA was extracted from 12-DAP kernels with the pericarp removed. The expression level was normalized against *ZmActin*. (E) Western blot analysis using antibodies against AOX (alternative oxidase) and α-tubulin as a sample loading control. (F-G) Morphological analysis of mitochondria in wild-type (F) and *Zmsmk3* (G) endosperms at 10-DAP. Mitochondria are indicated by the arrows. Scale bar = 0.5 µm.

To further investigate the assembly of respiratory complexes, mitochondrial proteins isolated from 12 DAP *Zmsmk3* and WT endosperm were analyzed by blue native polyacrylamide gel electrophoresis (BN-PAGE). The results showed that *Zmsmk3* mutants had a reduced accumulation of complex I (Figure 6A) and a significant reduction in complex I activity (Figure 6B), while the complex III was slightly increased, which might be as a complementary of the deficiency of complex I. Furthermore, we determined the steady‐state level of some subunits by western blotting. Surprisingly, a great difference between WT and *Zmsmk3* was revealed using an antibody against Nad6, a subunit of complex I. The accumulation of complexes IV and V subunits (Cox2 and AtpB) were insignificantly different between *Zmsmk3* and WT, whereas the accumulation of complexes III subunits (Cytc) was a little higher in *Zmsmk3* mitochondria than that in WT (Figure 6C). So the results showed that deficiency of splicing of *nad1* and *nad4* resulted in disordered assembly and activity of complex I.

We further investigated the morphology of the mitochondria in the endosperm at 10 DAP using transmission electron microscopy. It was discovered that the WT mitochondria showed normally folded cristae structures with distinct inner spaces (Figure 6F), whereas the cristae structure of *Zmsmk3* mitochondria was irregular (Figure 6G). Another alternative oxidation pathway was also analyzed by examining the expression of the three alternative oxidase (*AOX*) genes, *AOX1*, *AOX2* and *AOX3*. The expression of *AOX2* was much higher in the *Zmsmk3* mutants than that in the WT, and the expression levels of *AOX1* and *AOX3* were slightly increased (Figure 6D). Specific antibodies against the alternative oxidation proteins were used for western blotting. As a result, there was a dramatic increase in the levels of AOX proteins in *Zmsmk3* mutant (Figure 6E). These results indicated that the deficiency of ETC could cause a dramatic increase of the expression levels of *AOX* genes.

## Discussion

### ZmSmk3 is required for kernel development and seedling growth in maize

Here, we identified a candidate gene for *Zmsmk3* in maize, which encodes an mTERF protein required for the development of maize kernels and seedling growth. The kernels are composed of endosperm, embryo and pericarp. Endosperm is the main storage organ for nutrients, so the arrested endosperm development in *Zmsmk3* resulted in small kernels with reduced weights (Figure 1 and Figure S1). The differentiated endosperm is divided into four major cell types, namely the cells of the embryo surrounding region, the BETL, the aleurone layer, and the starchy endosperm (Olsen 2001). BETL is crucial to transport nutrients from maternal placenta to the endosperm in the development of kernels, and the absence of a properly formed BETL is correlated with reduced rates of grain filling and increased levels of seed abortion (Cai *et al.* 2017; Ren *et al.* 2017). Here, *ZmSmk3* was found to be highly expressed in the BETL of the endosperm (Figure 4e), suggesting that *ZmSmk3* is important for BETL development. Consistent with this finding, BETL differentiation was diminished in the *Zmsmk3* mutants (Figure 1), resulting in arrested nutrients enrichment in the *Zmsmk3* endosperm (Figure 2). This discovery was further supported by the down regulated expression of starch biosynthesis genes and reduced starch granules in the 12DAP endosperm of *Zmsmk3* mutants (Figure 2, Supplemental Table 1). The development of embryo proceeds in three stages in maize, namely the transition, coleoptilar, and late embryogenesis stages (Olsen 2001). In this study, we found that *ZmSmk3* was highly expressed in the SAM of embryos (Figure 4). The loss of *ZmSmk3* function caused the early halting of embryo development at the transition stage, resulting in an inflated transition stage embryo (Figure 1). However, part of the embryos of *Zmsmk3* mutant could germinate and grow into intact seedlings (Figure 1 and Figure S1) with lower germination rates and seedling survival rates, and the survived seedlings grew much slowly (Figure S1). These findings suggest that *ZmSmk3* is not only crucial for kernel development but also for the development of the seedlings. However, to finally validate the function of *ZmSmk3*, complementation and allelism tests would be adopted in the future research.

### ZmSmk3 affects complex I assembly by modulating the splicing of mitochondrial nad4 intron1 and nad1 intron4 in maize

NAD1 and NAD4 are the core components of complex I, which is located on the mitochondrial membrane (Klodmann *et al.* 2010; Meyer *et al.* 2011). There are three *trans*-introns and 0ne *cis*-intron in *nad1* and three *cis*-introns in *nad4*, and the proper splicing of them is crucial for their functions. Some PPR proteins have been reported to function as splicing factors of *nad1* and *nad4*. In *Zea mays*, the *defective kernel 2* (*dek2*) mutation reduced the splicing efficiency of mitochondrial *nad1* intron 1 and severely impeded complex I assembly (Qi *et al.* 2017). *Dek35* and *Emp11* encode PPR proteins that affected the *cis*-splicing of mitochondrial *nad4* intron 1 and *nad1* introns, respectively, which both influenced seed development in maize (Chen *et al.* 2017; Ren *et al.* 2017). In *Zmsmk3* mutants, the *nad1* intron4 and *nad4* intron1 could not be spliced properly, resulting in the decrease of mature transcripts of *nad1* and *nad4* (Figure 5A-C), while other mitochondrial introns in *Zmsmk3* were correctly spliced. So the results suggested that a single *Zmsmk3* mutation specifically affected the splicing of *nad1* intron4 and *nad4* intron1. RNA metabolism in plant mitochondria is a complex process that combines bacteria-like traits with novel features that evolved in the host cell, and is consequently regulated by families of nucleus-encoded factors (Hammani and Giege 2014). Various factors are required for the splicing and processing of mitochondrial introns in plants, including the PPRs (Barkan and Small 2014), mTERFs (Hammani and Barkan 2014), the regulators of chromosome condensation (Kühn *et al.* 2011), plant organellar RNA recognition proteins (Kroeger *et al.* 2009; Francs-Small *et al.* 2012), and chloroplast RNA splicing and ribosome maturation proteins (Zmudjak *et al.* 2013). To interpret the non-specificity of *ZmSmk3* to *nad4* intron1 and *nad1* intron 4, we assume that *ZmSmk3* may play a role in the recognition of precursor *nad4* and *nad1* mRNA and in the maintenance of the *nad4* and *nad1* conformation for intron splicing by cooperating with other factors. Consistent with previously reported phenotypes of the complex I-deficient mutants nuclear maturase 1 (nmat1) (Keren *et al.* 2012), nmat2 (Keren *et al.* 2009), and nmat4 (Cohen *et al.* 2014), in *Zmsmk3*, the defect mature transcripts of *nad1* and *nad4* affected the assembly and activity of complex I (Figure 6A-C), limiting ATP production. The loss of complex I function results in a defective ETC, which affects the respiratory metabolism and the inner mitochondrial structure (Qi *et al.* 2017). When the ETC is defective, alternative respiratory enzymes, the AOXs, are activated (Sabar *et al.* 2000). Plants with partial mitochondrial dysfunction can increase *AOX* expression to increase their tolerance of energy stress (Vanlerberghe *et al.* 2016). Similarly, our results found that the mitochondrial complex I was reduced in the mutant, and the expression of *AOXs* was dramatically increased in the *Zmsmk3* mutant (Figure 6D-E). Though it is known that ZmSMK3 functions in the splicing of *nad1* and *nad4*, its remains to be seen whether this factor have additional roles in organellar RNA metabolism.

### ZmSMK3 has the structural and functional divergence with its ortholog mTEFRF15 in Arabidopsis

According to the results, *ZmSmk3* is involved in the splicing of *nad1* intron4 and *nad4* intron1. However, in *Arabidopsis*, mTERF15, the ortholog of ZmSMK3, is required for mitochondrial *nad2* intron 3 splicing and complex I activity (Hsu *et al.* 2014). The structure comparison of the two proteins showed that there are 2 mTERF motifs in maize ZmSMK3, while there are 5 mTERF motifs in *Arabidopsis* mTERF15 (Figure 3B). More interestingly, the alignment of ZmSMK3 orthologs among 11 species showed that the homologous of ZmSMK3 in the monocotyledons was distinctly different from the homologous in dicotyledons in the number of mTERF motifs, which is exactly identical with the comparison between ZmSMK3 and mTERF15 (Figure 3B). However, the target introns in *Zea mays*, including *nad1* intron4, *nad2* intron3 and *nad4* intron1, showed considerable similarity to *Arabidopsis* (data not shown), suggesting that the introns were quite conserved during evolution between the two species. According to the results, the ZmSMK3 and mTERF15 could bind to different introns. Therefore, on one hand, the function of the mTERF proteins is conservative as both ZmSMK3 and AtmTERF15 were participate in intron splicing, on the other hand, the variation of their structure resulted in divergence in target intron splicing.
